# ECG Multi-Emotion Recognition Based on Heart Rate Variability Signal Features Mining

**DOI:** 10.3390/s23208636

**Published:** 2023-10-22

**Authors:** Ling Wang, Jiayu Hao, Tie Hua Zhou

**Affiliations:** Department of Computer Science and Technology, School of Computer Science, Northeast Electric Power University, Jilin 132013, China; smile2867ling@neepu.edu.cn (L.W.); 2202100964@neepu.edu.cn (J.H.)

**Keywords:** heart rate variability, emotion recognition, amplitude level quantization, feature extraction, logistic regression

## Abstract

Heart rate variability (HRV) serves as a significant physiological measure that mirrors the regulatory capacity of the cardiac autonomic nervous system. It not only indicates the extent of the autonomic nervous system’s influence on heart function but also unveils the connection between emotions and psychological disorders. Currently, in the field of emotion recognition using HRV, most methods focus on feature extraction through the comprehensive analysis of signal characteristics; however, these methods lack in-depth analysis of the local features in the HRV signal and cannot fully utilize the information of the HRV signal. Therefore, we propose the HRV Emotion Recognition (HER) method, utilizing the amplitude level quantization (ALQ) technique for feature extraction. First, we employ the emotion quantification analysis (EQA) technique to impartially assess the semantic resemblance of emotions within the domain of emotional arousal. Then, we use the ALQ method to extract rich local information features by analyzing the local information in each frequency range of the HRV signal. Finally, the extracted features are classified using a logistic regression (LR) classification algorithm, which can achieve efficient and accurate emotion recognition. According to the experiment findings, the approach surpasses existing techniques in emotion recognition accuracy, achieving an average accuracy rate of 84.3%. Therefore, the HER method proposed in this paper can effectively utilize the local features in HRV signals to achieve efficient and accurate emotion recognition. This will provide strong support for emotion research in psychology, medicine, and other fields.

## 1. Introduction

Emotions are innate physiological responses designed to maintain or restore homeostasis by altering the environment for more appropriate interactions [1]. Emotions are not only significant in the realm of social understanding and interaction, but they also trigger physiological mechanisms in order to proficiently recognize and address a diverse range of challenges and opportunities, thus contributing to our survival and growth [2]. Emotional experiences are closely linked to bodily perceptions, and they help direct attention to critical events, such as physical needs, potential threats, and social interactions. Emotional experiences play a role in coordinating behavioral and physiological responses during these critical occasions [3].

The autonomic nervous system (ANS) plays a significant role in conveying extensive data on emotional states, is tightly connected to emotional arousal, and at the same time, dominates the function of the lungs, heart, and numerous other organ systems [4]. Various mood-related physiological variables are often easily measured and thus detect altered states of the ANS [5]. Such changes usually occur unknowingly. For some stimuli and unconscious responses, the state of the response changes more rapidly than the conscious response. These physical aspects include facial expression, movement and gait, voice characteristics, electrocardiogram, electroencephalogram, electrooculogram, and galvanic skin response [6,7,8,9]. Of particular interest is the fact that the core area of our focus, the electrocardiogram (ECG), has been shown to have a strong correlation with emotional characteristics as well as with ECG waveforms. Numerous scholars have explored the feasibility and limitations of utilizing ECG signals for emotion detection [10]. Furthermore, recent developments in machine learning and deep learning have shown that emotion recognition systems can reliably derive information from ECG data [11]. Most sentiment recognition tasks involving machine learning or deep learning predominantly employ a fully supervised learning paradigm [12]. There are several limitations to this approach. First, in a typical fully supervised learning scenario, each classification or regression task requires re-training the model from scratch, which requires significant computational resources and time. In addition, features obtained from a fully supervised trained model are usually too task-specific to generalize well to other tasks. Finally, typical fully supervised learning usually requires training with large-scale manually labeled datasets, as small datasets usually lead to the performance degradation of deep networks. In this context, we will focus on extracting relevant features for different emotions and classifying them with the help of relevant machine learning algorithms to achieve reliable accuracy. There are many available features, among which the heart rate variability (HRV) signal is the most typical.

The autonomic nervous system (ANS) consists of the sympathetic and parasympathetic branches, working in harmony to oversee the functioning of target organs and tissues, ensuring the preservation of homeostasis [13]. Emotion is closely connected to these branches as they all provide nerve signals to the heart, with a particular emphasis on the AV node. This node collaborates with neuromodulation to facilitate the coordination of neurotransmitters, ensuring they work in harmony to regulate their functions and generate a distinct response to emotional conditions. Sympathetic overactivity may result in additional cardiac contractions or a rapid heartbeat. In contrast, parasympathetic responses to negative emotions are often influenced by olfactory or visual stimuli, which may result in a slowed heartbeat or even cardiac arrest [14]. The spectral examination of heart rate variability (HRV) is considered a non-intrusive technique to assess the balance between the two principal branches of the autonomic nervous system (ANS), which has been suggested for detecting and characterizing emotional states [15]. A set of guidelines has been formulated for evaluating, physiologically interpreting, and clinically applying resting heart rate variability (HRV), which includes the delineation of three distinct spectral components: an ultra-low-frequency (ULF) spectrum ranging from 0 to 0.04 Hz, a low-frequency (LF) spectrum covering the range from 0.04 to 0.15 Hz, and a high-frequency (HF) spectrum spanning from 0.15 to 0.4 Hz. HF band power is regarded as an indicator of parasympathetic activity, primarily attributed to respiratory sinus arrhythmia. The power within the low-frequency range is regarded as an indicator of both the sympathetic and parasympathetic activity of the heart. HRV serves as a mirror to the fluctuations in the autonomic nervous system, which, in turn, can mirror the accompanying emotional states.

Therefore, the purpose of this paper is to study the interconnection between emotions and HRV signals in ECG signals and to explore the differences in the HRV features between different emotions by extracting the relevant features of HRV signals, so as to recognize and classify high-dimensional emotions.

The paper is organized as follows: Section 1 presents the background of current research on emotion recognition; Section 2 introduces the current state of research; Section 3 presents the materials and methods, as well as the motivation for the study; Section 4 presents a detailed description of the computational process of the HER model; Section 5 presents the experimental analyses and the presentation of the results; Section 6 presents a detailed discussion; and Section 7 concludes the study.

## 2. Related Work

Presently, the primary focus in emotion recognition through ECG revolves around the utilization of classification algorithms alongside the selection of pertinent features and the scope of identifiable emotions. Machine learning algorithms have proven to be effective for emotion recognition based on ECG data. Axel and his team employed the wavelet scattering method for the extraction of unique characteristics from ECG data. This method facilitated the acquisition of signal features spanning varying time scales to assess their performance. The outcomes of this study demonstrated that the proposed feature-extraction and signal classification algorithm, within the realm of emotional dimensions, achieved an accuracy rate of 88.8% for arousal, 90.2% for valence, and an impressive 95.3% for two-dimensional classification [16]. Theekshana et al. presented a machine learning approach utilizing an ensemble learning method to identify core emotions, including anger, sadness, happiness, and the combination of happiness with electrocardiogram (ECG) data. They utilized spectral analysis as a novel approach to extract features and assessed the efficacy of a widely recognized collection of ensemble learners for emotion classification through a machine learning process. The ensemble learners exhibited a 10.77% enhancement in accuracy when compared to the top-performing individual biosensor-based model [17]. Yan et al. introduced the X-GWO-SVM approach to analyze sentiments from ECG data. They conducted single-subject cross-validation and achieved an impressive average accuracy rate of 95.93% when utilizing the WESAD dataset. This result demonstrates its superior reliability compared to previous implementations of supervised machine learning techniques [18].

Deep learning algorithms are commonly employed in the realm of emotion recognition as well. Pritam et al. devised a novel approach using a self-supervised deep multitask learning framework to detect emotions based on electrocardiogram (ECG) data. In this process, the convolutional layer remained fixed, while the dense layer was fine-tuned using labeled ECG data. Notably, this innovative solution yielded state-of-the-art results in classifying arousal, mood, emotional state, and stress across four distinct datasets [19]. Xu et al. introduced an emotion detection technique rooted in deep learning tailored for healthcare data analysis. Their approach incorporated a multichannel convolutional neural network to extract distinctive features from ECG data and textual content related to emotions, particularly for detecting emotional fatigue. Ultimately, this method amalgamated the features derived from multiple data sources to ascertain emotional states. The experimental findings demonstrated that the proposed model consistently achieved an accuracy rate exceeding 85% in the prediction of emotional fatigue [20]. Chen et al. introduced a novel approach to emotion recognition, which involved combining multiple sensory modalities using the Dempster–Shafer evidence theory. They utilized an SVM classifier to categorize EEG signal features. The results of the experiment demonstrated that the multimodal fusion model outperformed the unimodal emotion detection method, resulting in a significant increase in accuracy by 7.37% and 8.73% as compared to the emotion recognition model based on ECG signals [21]. Hammad et al. applied the PETSFCNN model in their research on emotion recognition, achieving an impressive maximum classification accuracy of 97.56%. They employed a deep neural network in combination with grid search optimization to enhance accuracy in classification tasks. The results of their study indicated that the suggested method surpasses existing techniques in precisely identifying emotions from ECG signals. This implies the possibility of utilizing it as an intelligent system for emotion recognition [22].

When it comes to selecting features for emotion recognition, HRV-related characteristics have gained widespread usage. Guo et al. conducted an analysis of ECG signals to extract heart rate variability (HRV) features, employing techniques encompassing frequency domain, time domain, statistical methods, and Poincaré. The HRV characteristics were later utilized for categorizing different emotional states, utilizing principal component analysis, and then employing a support vector machine to reduce the feature set. Notably, they achieved classification accuracies of 71.4% for distinguishing two emotional states (positive/negative) and 56.9% for classifying five emotional states [23]. Ferdinando et al. employed K-nearest neighbors (KNN) as a classifier in their study. They combined supervised dimensionality reduction using neighborhood component analysis (NCA) with feature-extraction techniques, which included capturing standard HRV features and statistical distributions of instantaneous frequencies. These methods were applied to address the classification of three distinct emotion and arousal categories. The findings suggested that, in most instances, integrating NCA resulted in a significant performance boost of 74% when compared to the absence of NCA in the implementation [24]. Singson et al. harnessed a ResNet-based CNN to analyze both facial expressions and physiological data, with a particular focus on heart rate variability (HRV) features. Their aim was to discern and validate emotions, achieving an accuracy rate of 68.42% through the analysis of ECG signals [25].

In previous studies, although it has been demonstrated that ECG-based emotion recognition is feasible, many works have been able to identify fewer kinds of emotions, mostly based on low-dimensional labels in the valence-arousal domain, which are not sufficiently refined or less diverse. Some of the recognition accuracy can also be limited. Therefore, emotion recognition based on ECG signals still needs further research.

## 3. Materials and Methods

### 3.1. Motivation

**Figure 1:**
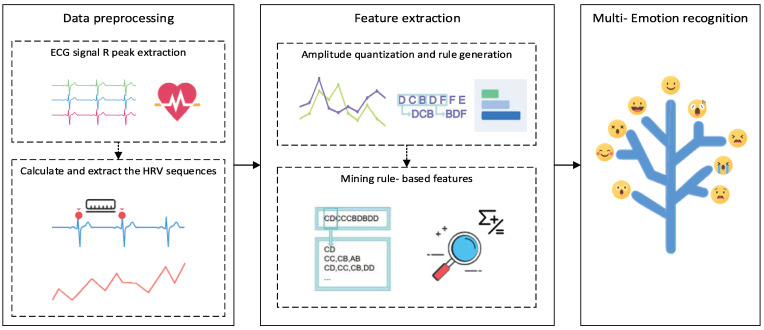
Multi-Emotion Recognition Flowchart.

In our study, combined emotional changes are better to express the real status of negative or positive emotions. Moreover, negative emotions combined with some kinds of positive emotions produce unexpected results, such as reinforced positive emotions. That means emotional expression is multidimensional, as are the changes in the autonomic nervous system between the main sympathetic nerve and the parasympathetic nerve. So, we performed multidimensional emotion recognition by collecting ECG signals and extracting HRV signals from a portable device to realize the accurate classification of emotions, as shown in Figure 1.

Based on the feature of the correlation of change patterns between HRV signals and the autonomic nervous system, we propose the HRV emotion recognition (HER) method to deeply explore the unique HRV signal fluctuation patterns between different emotions. We use algorithms such as rule extraction to explore the potential fluctuation patterns and other rules and also propose the amplitude level quantization (ALQ) feature-extraction method to extract and analyze the corresponding frequency band features of HRV signals of different emotion categories. Finally, we identify and classify them through the logistic regression classification algorithm (LR), which achieves good results and high accuracy.

### 3.2. Dataset

The DREAMER dataset was used in the experiments, which is a multimodal dataset consisting of EEG and ECG signals captured when emotions are elicited by audiovisual stimuli. Self-ratings of the emotional state after each stimulus were also captured, including the dimensions of valence, arousal, and dominance. All data were collected using an off-the-shelf, cost-effective wireless wearable device; for details on the DREAMER dataset, see [26]. The confirmed categorization results of this database on mood, arousal, and dominance are very close to those achieved by other databases using expensive, non-portable, medical-grade devices.

### 3.3. HRV-Based Emotion Recognition Model

**Figure 2:**
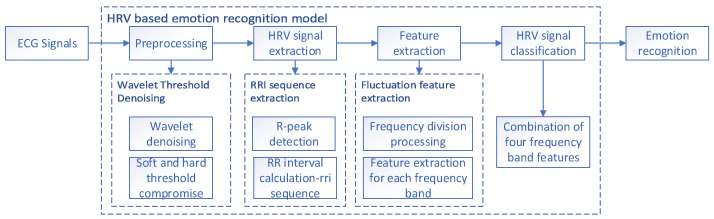
General architecture for computational models of emotion recognition.

To further explore the relationship between HRV responses and emotions, we objectively mapped emotions onto a valence-arousal two-dimensional domain using the emotion quantification analysis (EQA) method proposed in our previous work [27]. Since the previous work was based on EEG signals for emotion recognition, we utilized multimodal datasets with both EEG and ECG signals and used the EEG signals as an over-signal for the quantification of ECG signal labels to improve their accuracy. We propose the HRV emotion recognition model (HER model) to enhance the precision of emotional state detection. Figure 2 illustrates the general framework. First, data preprocessing is carried out for wavelet denoising, then extraction utilizes the R-peak extraction algorithm to extract and compute the HRV signal; this is followed by frequency band decomposition and feature extraction, and finally recognition classification.

## 4. HRV Emotional Recognition Model (HER Model)

**Figure 3:**
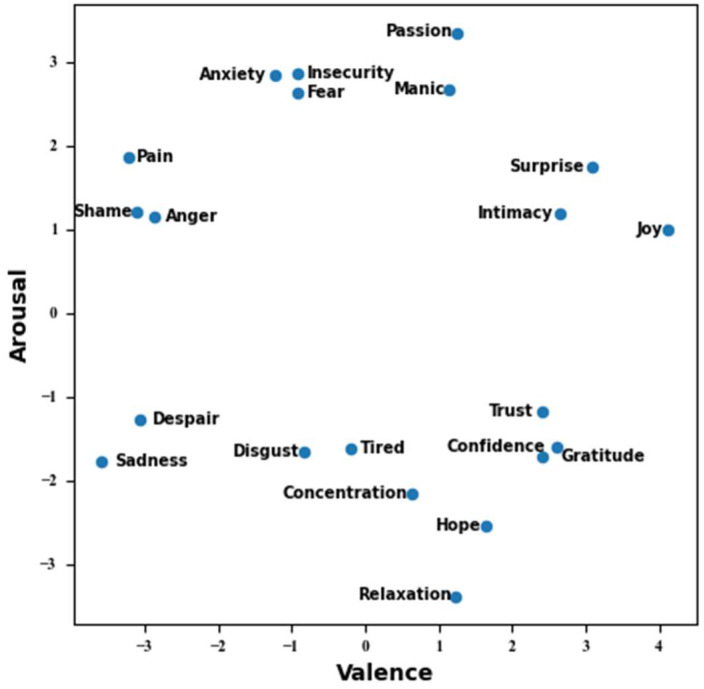
Emotion mapping within the valence-arousal domains.

Our proposed HER model identifies 17 emotions with high dimensionality based on disaggregating emotions to objectively detect changes in emotions. We have previously studied the core emotion types, which are trained on the basis of a large amount of Weibo social big data. The number variety reached more than 20 types, and these core emotions were calculated by similarity and perspective [28]. In addition, we tested these 20 emotion types by EEG signals, and all of them showed significant arousal rates [27]. Simultaneously, the trustworthiness of the emotion assessments was affirmed by aligning emotional content in EEG signals with music audio signals and delving further into potential connections between emotional and music responses [29]. Figure 2 illustrates how these emotions are distributed within the arousal value model, which reflects the outcomes derived from semantic computation. We generated these labels using our proprietary emotion dictionary, employing semantic similarity measurements across over 20 emotion descriptors, each of which encompasses a range of more than 10,000 emotional variations. This semantic method of computation is also key to improving the recognition accuracy. Then, we refined the labels to 17 emotions according to their valence-arousal labels using the EQA method, including surprise, sadness, anxiety, passion, joy, shame, hope, tired, fear, disgust, anger, gratitude, intimacy, trust, pain, confidence and relaxation. Four of these emotions were not found in the DREAMER dataset. Figure 3 illustrates how these emotions are distributed within the arousal-value spectrum.

### 4.1. Data Denoising

Since the common denoising methods can only remove information frequencies with specific cutoff frequencies in the ECG signal, we use the DWT-based wavelet denoising method to pre-process the ECG signal to remove noise, artifacts, external interference, etc. The wavelet denoising method does not need to specify the cutoff frequency and sampling frequency, which is more convenient and reliable. Here we choose the “sym8” wavelet base and perform five-layer wavelet decomposition. In the pre-processing process, we chose a compromise between hard and soft thresholding, which is more suitable for ECG denoising and is better for retaining R-peaks and other key parameter information, which is beneficial for subsequent calculations. The threshold selection is calculated as follows:

(1) $$\theta =\delta \times \sqrt{2\times \log_{e}(H)}$$

In this context, H represents the length of the signal, θ signifies the threshold value, and δ stands for the noise’s standard deviation coefficient. The noise standard deviation coefficient is calculated as follows:

(2) $$\delta =(1/0.6745)\times \frac{\sum_{i=1}^{n}\left|w_{i}\right|}{n}$$

The $w_{i}$ sequence represents the detailed coefficient sequence decomposed by the “sym8” wavelet basis, and δ represents the noise standard deviation coefficient. The denoising equation is as follows:

(3) $$\gamma =\left\{ \begin{array}{l} \varphi_{w}\times \left|w-0.5\times \theta \right|,\left|w\right|\geq \theta \\ 0,\left|w\right|<\theta \end{array}\right.$$

In the context of wavelet coefficient processing, γ represents the wavelet coefficients post-noise reduction, while w signifies the wavelet coefficients prior to noise reduction. Additionally, $\phi_{w}$ stands for the symbolic evaluation formula applied to these wavelet coefficients, as follows:

(4) $$\phi_{w}=\left\{ \begin{array}{l} 1,\;w>0\\ 0,\;w=0\\ -1,\;w<0 \end{array}\right.$$

### 4.2. HRV Signal Extraction

**Figure 4:**
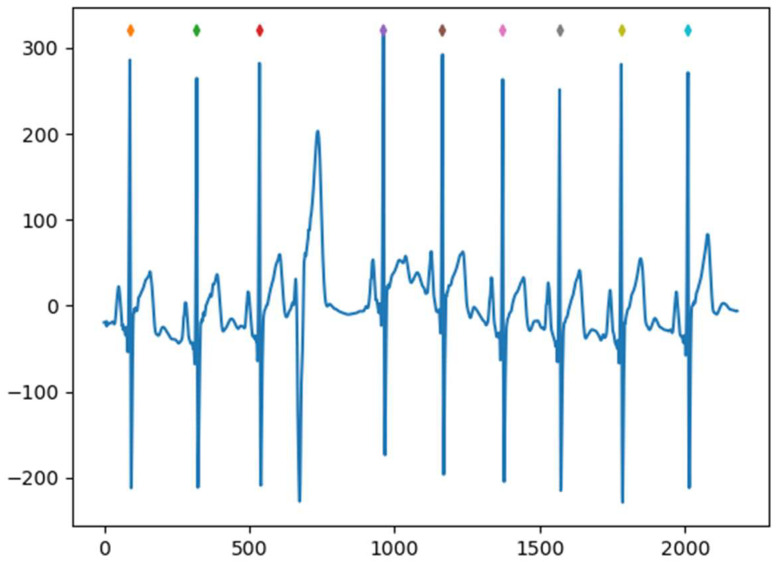
R-peak identification in QRS wave groups.

HRV, often assessed by analyzing changes in RR intervals derived from electrocardiogram (ECG) recordings, pertains to the fluctuations observed in consecutive heartbeats. The normal waveform of the ECG signal consists of the most prominent characteristic waves such as the T-wave, QRS-wave cluster, and P-wave, which reflect the activity state of the heart. Among them, the QRS wave cluster reflects the potential changes during the depolarization of ventricular muscle, while the RR interval feature, consisting of two adjacent R peaks, is mostly used for mood recognition. In the process of HRV signal extraction, accurately recognizing the consecutive R peaks in the ECG signal is of paramount importance, which provides a reliable basis for the subsequent calculation. In summary, the extraction of the HRV signal includes two parts: firstly, the R peaks of ECG signal are extracted, and then the time distance between adjacent R peaks is calculated to finally obtain the HRV signal. Among them, the results of identifying R peaks are shown in Figure 4.

### 4.3. HRV Signal Extraction Algorithm

The parameters of the HRV signal extraction algorithm are shown in the following figure (Table 1):

In the process of detecting R peaks in QRS wave groups for ECG signals, the signals have certain measurement errors, resulting in an incomplete display of QRS wave groups and thus the inaccurate identification of R peaks. The R-peak detection algorithm proposed in this paper can filter out the QRS wave groups that do not match a specific QRS wave group according to a specific QRS filter—as shown in Figure 3, a case of non-compliant R-peaks was not detected. In the subsequent interval calculation, the temporal distance of the non-conforming adjacent R peaks is filtered according to the threshold value, which makes the extracted HRV signal more consistent with the actual situation.

R-peak detection was performed by calculating the intercorrelation, where the QRS filter was selected with the following equation:

(5) qrs_filt=sin(t), t∈(1.5π,3.5π)

where qrs_filt is the filter function. The similarity is calculated as follows:

(6) simi=correlate(WEC, qrs_filt)

simi is the similarity and the correlate function is a function used to calculate the correlation value of two one-dimensional sequences. The formula for discriminating by a specific threshold value and extracting the R-peak interval values is as follows:

(7) $$\mathrm{PK}=\left\{ \begin{array}{l} \mathrm{index}(WEC),\;\mathrm{simi}>TH\\ 0,\;simi\leq TH \end{array}\right.$$

PK is the R-peak interval value, which is the interval where the R-peak time value is located. index function is the time label of the returned series. Based on the R-peak interval values, the median is used to determine the final R-peak, and the formula is as follows:

(8) PG=median(PK)

where PG is the final R peak and median is the median finding function. In the PG sequence, the difference between two adjacent values is found in turn to obtain the HRV signal, with the following equation:

(9) $$\mathrm{RRI}=x_{i+1}-x_{i},\;x\in PG,\;i\in (0,\;n-1)$$

The details of the HRV signal extraction algorithm are shown below (Algorithm 1):
**Algorithm 1:** HER Model—HRV Signal Extraction Algorithm**Input:**EC**Output:**RRI sequence1: **Begin**2: import EC to Python3: X ← EC; *TM* ← Soft and hard threshold compromise; *WAVE* ← sym8; *WL* ← *5*4: **for** each of EC **do**5:  WEC = wave_noising (X, *TM*, *WAVE*, *WL*)6: **end for**7: X ← WEC; *QRSF* ← sin (linspace(1.5*${}_{\pi }$, 3.5*${}_{\pi }$, 15)); *TH* ← 0.37: **for** each of WEC **do**8:  similarity = correlate (X, *QRSF*)9:  **if** similarity > *TH*
**then**10:   PK += WEC.index11:   PG += median (PK)12:  **end if**13:  **for** i = 0, i < length (PG), i++ **do**14:   RRI += [PG (i + 1) − PG(i)]15:  **end for**16: **end for**

### 4.4. Rule Extraction

After the HRV signal is extracted, because its different frequency bands are related to different autonomic nervous system indicators—as an example, the parasympathetic nervous system (PNS) is correlated with the higher frequency range (0.15–0.5 Hz), whereas the sympathetic nervous system (SNS) is connected to the lower frequency range (0.04–0.15 Hz) of the HRV signal—we have further processed the HRV signal here. Using wavelet packet frequency band decomposition technology, it is subdivided into four different frequency bands, namely LF, HF1, HF2, and HF3. The specific frequency band range is shown in Table 2. After frequency band decomposition, we performed amplitude level quantization (ALQ) feature extraction for each frequency band signal, as we believe that for similar emotions, their amplitudes have similar transformation methods. Firstly, we conducted a hierarchical quantization of the amplitude and classified it into “F” levels based on the maximum amplitude of each band as a whole; divided into “E” levels between 10% and 20%; divided into “D” levels between 20% and 40%; divided into “C” levels between 40% and 60%; divided into “B” levels between 60% and 80%; and divided into “A” levels between 80% and 100%. According to the above division rules, we quantified and extracted each frequency band to obtain the corresponding level sequence.

According to what has been described above, in the valence-arousal domain, the emotion label of the HRV signal is quantified into the one emotion that is closest to it by calculating the distance with the emotion label in the emotion map. Thus, the amplitude quantization sequences of each of the 17 emotion categories are obtained. In order to count the most representative rank arrangement combinations of each emotion, we use the sliding window, and the lengths of the sliding window are taken as 2, 3, 4 and 5 in turn. According to the different lengths, multiple combining sequences of each band of the heart rate variability signal are computed sequentially, so as to further extract the combining sequence rules for each emotion.

**Figure 5:**
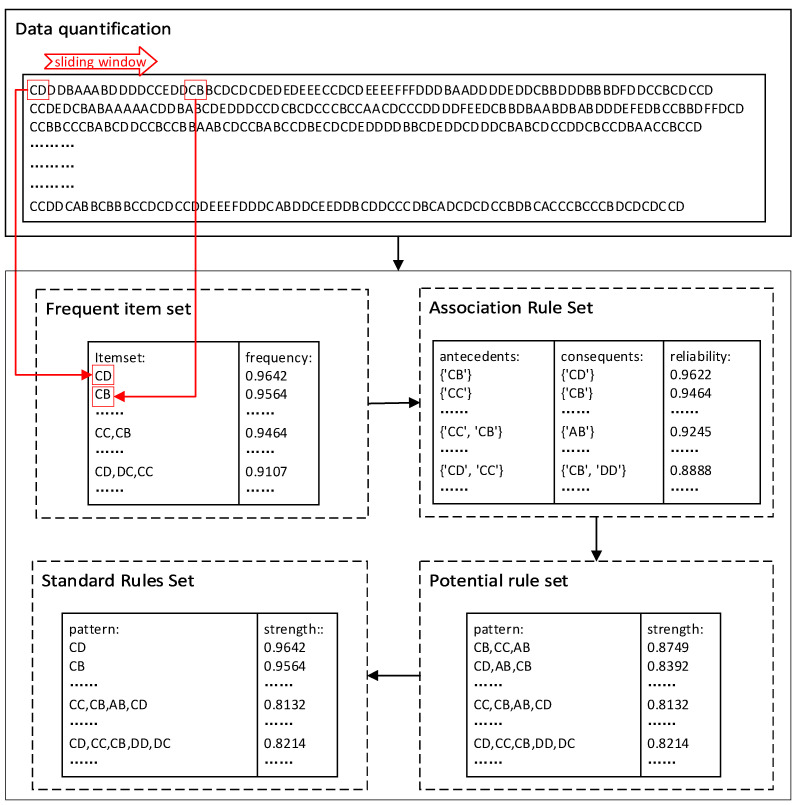
Rule Extraction Example Diagram.

In the process of calculating the rules of combination sequences, the FP-growth algorithm in association rules is used to mine the set of frequent items. First, the frequency of occurrence of each combination sequence in each emotion is calculated and the lower frequency ones are discarded. Then, the association rule set is generated for the retained combination sequences and the reliability of the newly generated combinations is calculated, again discarding the ones with lower reliability, whose reliability is considered as the strength value of each combination rule. Finally, the correlation value of two neighboring permutations in the association rule set is calculated, which refers to the possibility of both existing at the same time. If the correlation value is high and the former combination exists in the existing rules, the two neighboring combinations are categorized into the potential rule set, whose strength value is the product of the reliability of the former combination and the correlation value of the two, which, along with the set of frequent items, forms the standard rule set. An example diagram of this process is shown in Figure 5, where the length of the sliding window is taken as 2 units of length. From the figure, it can be seen that the standard rule set has a high-intensity value, which represents its applicability.

**Figure 6:**
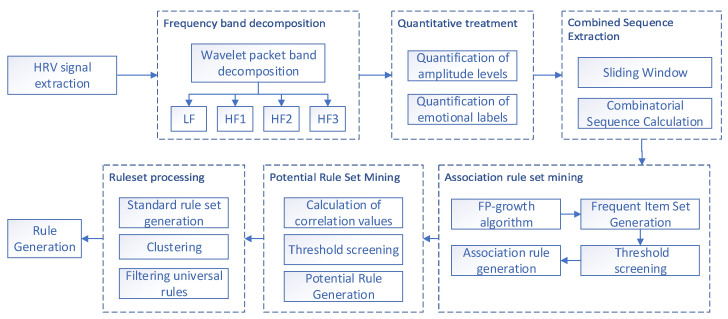
Rule extraction workflow.

Since there are sequence combination rules common to multiple emotions, at this point, such rules with multiple emotion labels will no longer be applicable in the subsequent matching work. Therefore, the rule set needs to be further processed, and we combine the rules of all emotions together and perform clustering to find out all emotion labels common to each combination rule, thus filtering out the sequence combinations common to all emotions, i.e., filtering out the universal rules. The workflow of rule extraction is shown in Figure 6.

### 4.5. Rule Extraction Algorithm

The rule extraction algorithm parameters are presented in Table 3.

The wavelet packet decomposition technique was used to perform the frequency band decomposition with the following equation:

(10) WRRI=reconstruct(WaveletPacket(RRI))

where RRI is the HRV signal sequence, WRRI is the signal sequence after band decomposition, WaveletPacket and reconstruct functions are the band decomposition and reconstruct functions of wavelet packet, respectively. The formula for quantization of the amplitude level is as follows:

(11) $$\mathrm{AQ}=\left\{ \begin{array}{l} A,\;\left|\mathrm{RA}\right|\in (0.8\times \mathrm{Amax},\;\mathrm{Amax})\\ B,\;\left|\mathrm{RA}\right|\in (0.6\times \mathrm{Amax},\;0.8\times \mathrm{Amax})\\ C,\;\left|\mathrm{RA}\right|\in (0.4\times \mathrm{Amax},\;0.6\times \mathrm{Amax})\\ D,\;\left|\mathrm{RA}\right|\in (0.2\times \mathrm{Amax},\;0.4\times \mathrm{Amax})\\ E,\;\left|\mathrm{RA}\right|\in (0.1\times \mathrm{Amax},\;0.2\times \mathrm{Amax})\\ F,\;\left|\mathrm{RA}\right|\in (0,\;0.1\times \mathrm{Amax}) \end{array}\right.$$

AQ is the sequence after amplitude level quantization, RA is the amplitude value of the wave crest and trough, and Amax is the absolute value of the maximum amplitude value in the sequence. The formula for mining frequent item sets using the frequent pattern growth algorithm is as follows:

(12) FI=fpgrowth(AQ)

where FI is the frequent itemset and the fpgrowth function is used to mine the frequent itemset. The calculation frequency is the core calculation formula:

(13) $$\mathrm{frequency}(X)=\frac{Transactions\;containing\;X}{Total\;transactions}$$

where *frequency(X)* denotes the support level of itemset *X*. *Transactions containing X* denotes the number of transactions containing itemset *X*. *Total transactions* denotes the total number of transactions. The formula for mining association rules is as follows:

(14) PR=association_rules(FI)

where PR is the potential rule set, the association_rules function is used to mine the association rule set in the frequent itemset. The formula for calculating *reliability* is shown below:

(15) $$\mathrm{reliability}(X\to Y)=\frac{\mathrm{frequency}(X\cup Y)}{\mathrm{frequency}(X)}$$

where: *reliability(X* → *Y)* denotes the reliability of rule *X* → *Y*. *frequency(X∪Y)* denotes the frequency of transactions that contain both item sets *X* and *Y*. *frequency(X)* denotes the support of item set *X*. The formula for mining potential rule sets is as follows:

(16) RU=mine(FI, PR)

where RU is the merged ruleset, and the mine function is used to mine the potential rule set in the frequent item set and the association rule set and generate the standard rule set.

The details of the rule extraction algorithm are shown below (Algorithm 2):
**Algorithm 2:** HER Model—Rule Extraction Algorithm**Input:**RRI**Output:**RU1: **Begin**2: import RRI to Python3: X ← RRI, *WAVE* ← db10, *WL* ← 124: **for** each of RRI **do**5:  WRRI = reconstruct (WaveletPacket (X, *WAVE*, *WL*))6:  The WRRI is divided into amplitude classes to obtain the class sequence AQ7: **end for**8: Clustering of AQ’s labels9: **for** each of labels **do**10:  FI = fpgrowth (AQ)11:  PR = association_rules (FI)12:  RU = mine (FI, PR)13:  **for** each rule of RU **do**14:   **if** rule has all the emotional labels **then**15:    RU −= rule16:   **end if**17:  **end for**18: **end for**

### 4.6. Extraction of Amplitude Level Quantitative Features

**Figure 7:**
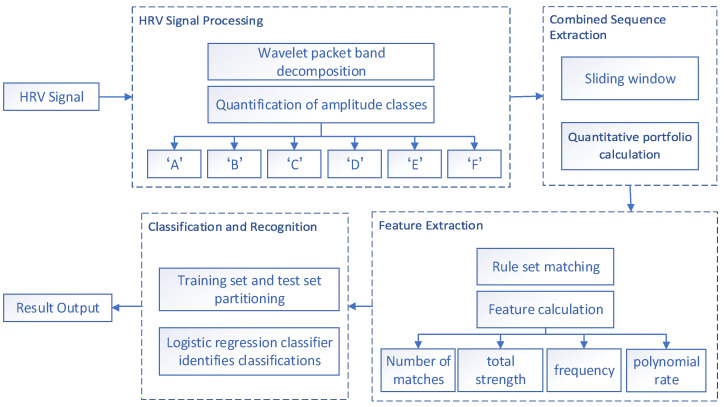
Amplitude level quantitative feature-extraction workflow.

While conducting emotion recognition through physiological signals, the pivotal stage lies in feature extraction, and a significant portion of research is dedicated to this aspect. In this paper, after extracting the rule set of all emotions, the amplitude level quantization (ALQ) features are extracted based on this rule set, and this feature-extraction method provides a good basis for the subsequent high recognition accuracy. Since the HRV signals of each frequency band are independent of each other, the obtained amplitude level sequences are also different, so the sliding window is also used to extract several combinations of sequences from each HRV signal for the amplitude level sequences in each frequency band and match them with the rule set. For each of the four frequency bands of the HRV signal, four features of the ALQ were extracted, which are the number of matches, total strength, frequency, and polynomial rate. After feature extraction, a total of 16 features in four frequency bands are placed into a logistic regression classifier for training and recognition. The flow of the related work is shown in Figure 7.

### 4.7. Amplitude Level Quantitative Algorithm

The parameters of the amplitude level quantization algorithm are shown in the following figure (Table 4):

MA is the number of times each quantized sequence matches the rule set, which is calculated as follows:

(17) $$\mathrm{MA}=\left\{ \begin{array}{l} \mathrm{MA}+1,\;{\mathrm{AQ}}_{i}=RU_{i}\\ \mathrm{MA},\;AQ_{i}\neq RU_{i} \end{array}\right.$$

where AQ is the new combined sequence of amplitude rank quantization sequences extracted by sliding window and RU is the rule set. The total strength TC value is calculated as follows:

(18) $$\mathrm{TC}=\left\{ \begin{array}{l} TC+{\mathrm{CO}}_{i},\;{\mathrm{AQ}}_{i}=RU_{i}\\ TC,\;{\mathrm{AQ}}_{i}\neq RU_{i} \end{array}\right.$$

where ${\mathrm{CO}}_{i}$ is the strength value of each rule in the rule set. The frequency FR by using the following formula:

(19) $$\mathrm{FR}=\frac{MA}{LE}$$

where LE is the total number of new sequences extracted by the sliding window for the rank quantized sequences. The polynomial ratio PR can be determined using the following mathematical expression:

(20) $$\mathrm{PR}=\frac{\sum_{i=1}^{L\max}MNl_{i}-MNl_{1}}{MA}$$

where ${\mathrm{MNL}}_{i}$ is the number of matched combinatorial sequences of length *i* and Lmax is the maximum length of the combinatorial sequence. After the extraction of the features is finished, they are put into a logistic regression classifier for training and recognition tests with the following equation:

(21) Acc=LogisticRegression(MA, TC, FR, PR)

where Acc is the final recognition accuracy and LogisticRegression function is used for logistic regression for recognition classification.

Details of the amplitude level quantization algorithm are shown below (Algorithm 3):
**Algorithm 3:** HER Model—Amplitude Level Quantitative Algorithm**Input:** AQ, RU**Output:** Acc1: **Begin**2: import AQ, RU to Python3: **for** each sequence of AQ **do**4:  **for** i = 0, i < length (sequence), i++ **do**5:   **for** j = 0, j < length (RU), j++ **do**6:     **if** sequence(i) = RU (j) **then**7:      MA += 18:      TC += CO(i)9:     **if** length(sequence (i)) != 1 **then**
10:      Add the sequence (i) to the MNL set11:     **end if**12:    **end if**13:   **end for**14:  **end for**15: LE = length (AQ)16: FR = MA/LE17: PR = length (MNL)/MA18: **end for**19: model = LogisticRegressionTrain (train_label, train(MA, TC, FR, PR))20: Acc = LogisticRegressionPredict (model, test_label, test(MA, TC, FR, PR))

## 5. Experiment and Results

The computer utilized for the experiment is equipped with a Core8750H CPU with a main frequency of 2.2 GHz and the software is PyCharm Community Edition 2020. The programming environment is Python 3.8.

### 5.1. Dataset

In our experiments, the dataset we chose is the DREAMER public dataset [26], a multimodal dataset with EEG and ECG signals, as detailed in Section 3.2. The dataset is composed of the ECG signals acquired with a frequency of 256 Hz using the SHIMMER sensor, which contains 23 groups with over 21.09 million volumes. Moreover, 80% of the dataset is partitioned into the training set and 20% into the test set.

### 5.2. HER Model Test

**Figure 8:**
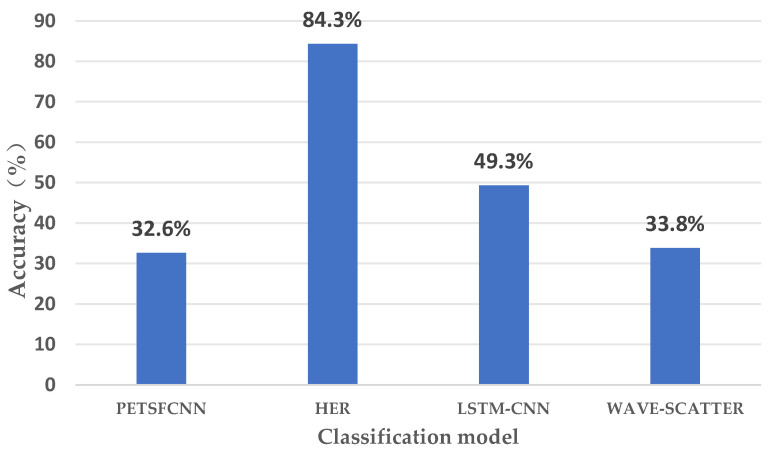
Accuracy results of classification models.

After conducting the experiments, we tested the HER model, and the results showed that it was effective for the classification of 17 dimensional emotions. We compared the experiments using the PETSFCNN model [22], the WAVE-SCATTER model [16] and the LSTM-CNN model [30]. Among them, the HER model’s impressive performance is evident, achieving an 84.3% accuracy rate, as illustrated in Figure 8 with detailed experimental findings.

**Figure 9:**
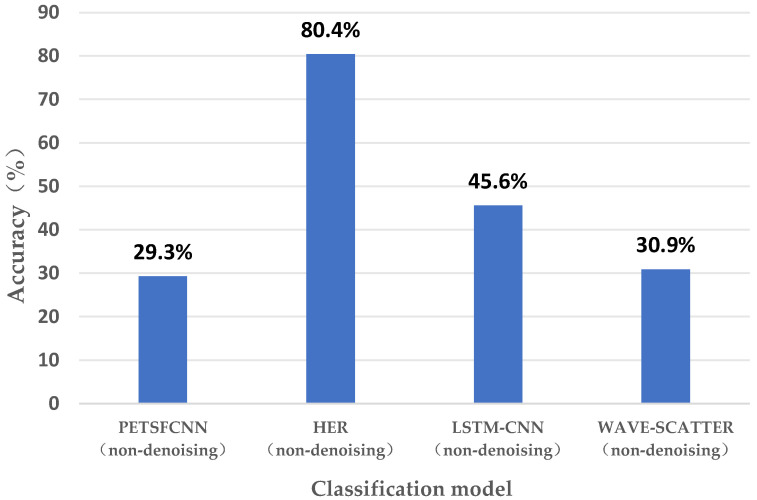
Accuracy results of the classification model for the raw data.

Meanwhile, in order to detect the corresponding processing effect of the denoising algorithm, we utilized the non-denoised raw data to conduct relevant experiments, and the experimental effects of the four models are shown in Figure 9. As can be seen from the figure, in the original data, the maximum accuracy of the HER model is only 80.4%, and compared with Figure 8, the accuracy is lower than that of the denoising process, and the other models cannot reach this accuracy after denoising. This shows that extracting the HRV signal in the non-denoised raw data is unreasonable, which will lead to the existence of a large bias, seriously affecting the subsequent recognition process. The effect of the denoising algorithm mentioned in this paper is very significant in terms of the accuracy comparison.

**Figure 10:**
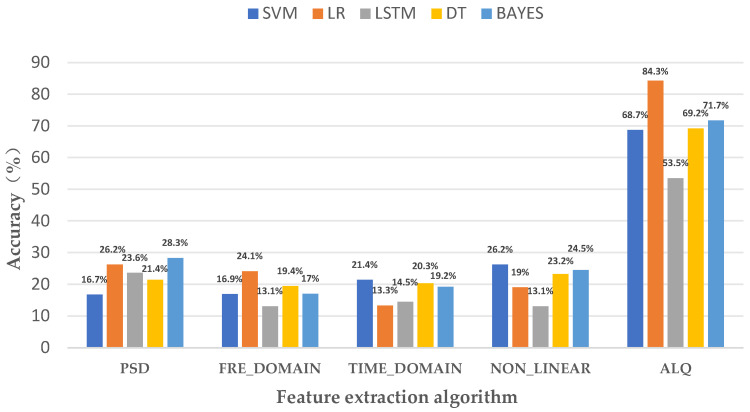
Classification results of multiple feature-extraction models.

The essence of the HER model lies in the ALQ feature-extraction approach, and after extracting a total of 16 features in 4 frequency bands using this feature-extraction method, we applied other traditional feature-extraction models for feature-extraction algorithm comparison experiments: power spectral density (PSD) features, time domain features, nonlinear features, and frequency domain features. We used four other classification algorithms for classification comparison experiments, which are the long short-term memory network (LSTM) model, support vector machine (SVM) model, decision trees (DT) model, and Bayesian networks (BAYES) model to assess the impacts of various classification algorithms on ALQ feature-extraction methods. The results show that the highest power spectral density feature-extraction model is 28.3%, the highest frequency domain feature-extraction model is 24.1%, the highest time domain feature-extraction model is 21.4%, the highest nonlinear feature-extraction model is 26.2%, and the highest ALQ method is 84.3%, which is the best result obtained by logistic regression model (LR), while the accuracy of SVM model is 68.6%, the accuracy of DT model is 69.2%, the accuracy of BAYES model is 71.1%, and the LSTM model has the worst result, with an accuracy of 53.4%. This indicates that the LR classification model is more suitable for classifying and identifying multi-band features. In the comparison experiments, the LR classification model demonstrates strong performance in both power spectral density and frequency domain feature models, as evident from the results, which also indicates that the LR classification model is more suitable for the classification recognition of frequency-related features. The ALQ feature-extraction algorithm proved to be more effective compared to the traditional feature-extraction model. Figure 10 illustrates the particular findings obtained from the experiment.

**Figure 11:**
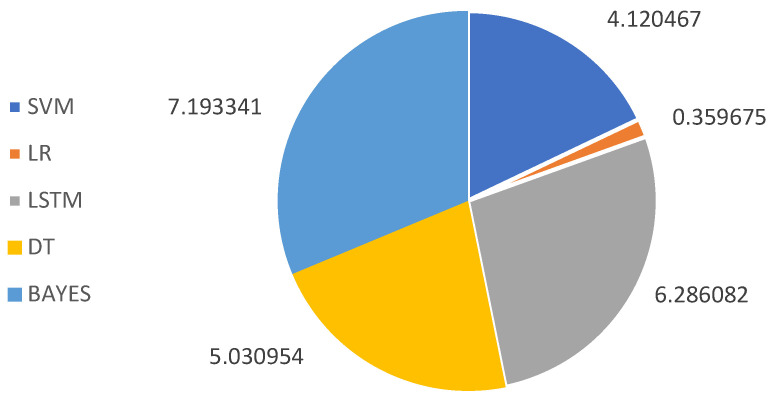
Running time comparison of different classification algorithms.

We also conducted comparative experiments on the running time of the five algorithms of the ALQ feature-extraction method, in which the LR algorithm has the shortest time and proves to be more efficient, as shown in Figure 11, with units accurate to the microsecond level.

**Figure 12:**
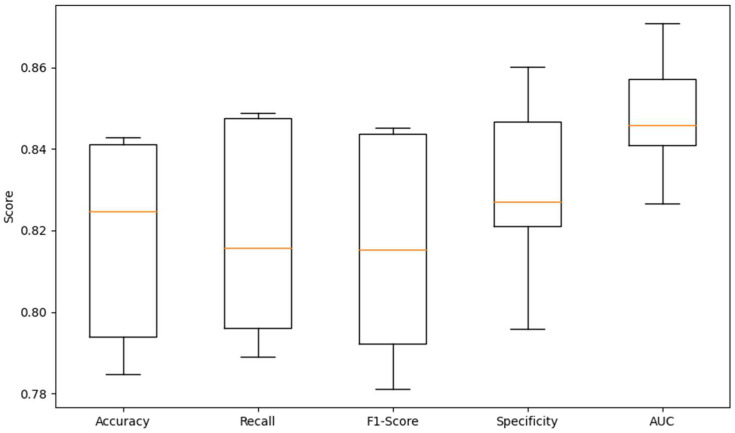
Box-whisker plot for 5-fold experimental design.

After obtaining the accuracy, other evaluation metrics of the HER model and the comparison model, such as F1 score, precision recall and specificity, were also analyzed, as shown in Table 5. We used a five-fold experimental design to debug the ratio of the training to the test set and obtained the values of the corresponding metrics with the box-whisker plot shown in Figure 12. The HER model achieves an F1-score of 0.8437, a Recall score of 0.8487, a Specificity score of 0.8602, and an AUC score of 0.8708, respectively. As evident from the results, this demonstrates its remarkable effectiveness.

**Figure 13:**
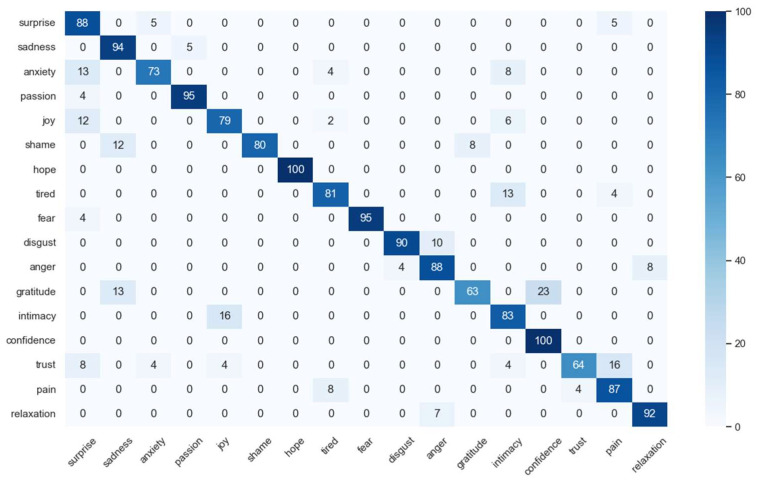
Confusion matrix for 17 emotions using the DREAMER dataset.

The assessment of the HER model’s recognition performance is based on the utilization of a confusion matrix, as depicted in Figure 13. It was found that six of the emotions achieved more than 80% recognition accuracy, namely “surprise”, “shame”, “tired”, “anger” and “intimacy”. Seven emotions achieved more than 90% accuracy, namely “sadness”, “passion”, “hope”, “fear”, “disgust”, “confidence” and “relaxation”. It can be seen that the HER model achieves higher accuracy classification, which fully reflects the recognition classification performance of this model. However, the analysis reveals a significant recognition discrepancy between “gratitude” and “confidence”, potentially attributed to their relatively low semantic distance within the EQA methodology, which is computed at approximately 0.22. The accuracy of gratitude is only 63%, while 23% probability is predicted as “confidence”. This also indicates that the two emotions are relatively similar. The recognition results for “hope” and “confidence” may be over-fitted because of the small amount of raw data for these two emotions.

## 6. Discussion

The classification results of this experiment show that the recognition of emotions using the HRV signal of ECG is very reliable, and the extracted ALQ features achieve high accuracy in the recognition of emotions in 17 categories. This indicates that the R-peak extraction algorithm mentioned in this paper is more suitable for the HRV signal feature extraction of ECG signals, and it also provides a reliable basis for obtaining high accuracy in this experiment. Another key point is that the rule sets of different emotions are extracted by using association rules, and the extracted rule sets possess the difference between different emotion categories, which promotes the good performance of the ALQ feature-extraction method; this point is also very important. Also, for the results related to the proposed comparison experiments, it is very useful to perform a multi-band decomposition of the HRV signals of ECG signal species, where different frequency ranges correspond to different physiological indicators. The logistic regression (LR) classification algorithm is more suitable for classifying and identifying multi-band features, especially for multi-classification identification, and it has the shortest time and is most efficient compared to other classification algorithms.

The capacity to categorize 17 emotional types based on HRV signals underscores the reflection of the human autonomic nervous system’s activity level and its intimate connection with emotional states. Due to the non-invasive and easy-to-use nature of HRV signal acquisition, this research holds significant practical implications and is poised to have a crucial impact across the realms of healthcare, psychological therapy, and human–computer interfaces.

In our experiments, the accuracy in detecting the EEG signal associated with the emotion “gratitude” was notably low. This can be attributed to the close semantic proximity between “gratitude” and “confidence”, which often leads to misinterpretation. Analyzing their positions within the two-dimensional emotional model, we observed their significant proximity, resulting in frequent confusion between these two emotions.

The HRV signal and its related features extracted in this paper can realize high-performance recognition and classification, and its main core advantage lies in the following: at the theoretical level, HRV is closely related to the response patterns of the autonomic nervous system species of the main sympathetic nerve and the parasympathetic nerve, and based on this, the advantage of the ALQ algorithm proposed in this paper is to be able to excavate specific fluctuation patterns between each emotion and to decompose the fluctuation patterns into various frequency ranges. Moreover, because the HRV signals in different frequency ranges represent the change patterns of the autonomic nervous system when generating different emotions, the use of these differential frequency band characteristics can achieve high-dimensional emotion classification, which has been proved to be effective by the experimental results. At the application level, the HER model proposed in this paper can be used for the portable detection of emotions by using HRV and does not require complex signal acquisition equipment, which can be realized by using HRV. It does not require complex signal acquisition equipment to realize high-precision emotion recognition, which reflects its convenient and fast application characteristics and is one of its advantages compared with other emotion detection methods.

## 7. Conclusions

This study employed ECG-derived HRV signals for the classification and analysis of a variety of emotions. The experimental findings in the domain of HRV signal-based emotion recognition indicated the successful identification of 17 distinct emotions. Moreover, the research involved analyzing the frequency band decomposition range to assess its utility in emotion classification. Consequently, the HER model demonstrated strong performance in semantically analyzing ECG-derived HRV sentiments, achieving an average accuracy rate of 84.3%. This underscores the effectiveness of the proposed frequency band decomposition range in emotion identification.

Future research endeavors will delve deeper into investigating the correlation between the assessment of HRV signals and emotions, with the aim of enhancing the precision and consistency of emotion categorization. Concurrently, we can explore the impact of incorporating HRV signals alongside other physiological indicators on the recognition of emotions, thus bolstering the precision and dependability of emotion recognition. This will have wide application prospects. In the medical field, using HRV signals for emotion recognition can help doctors better diagnose the emotional state of patients and guide the formulation of treatment plans. Leveraging HRV signals in the realm of human–computer interaction can lead to an enhanced user experience and more effective human–computer interaction outcomes. In addition, emotion recognition can also be applied to smart homes and psychological counseling to provide more convenient services to users.

## Figures and Tables

**Table 1:** Notations for HRV signal extraction algorithm.

Parameters	Definition
*EC*	Raw ECG signal
*TM*	Threshold method
*WAVE*	Wavelet basis functions used
*WEC*	ECG signal after denoising
*WL*	Wavelet decomposition level
*QRSF*	Filters used
*TH*	Similarity threshold
*PK*	Timestamp with similarity greater than the threshold
*PG*	Detected R peak group
*RRI*	HRV signal sequence

**Table 2:** HRV signal frequency band decomposition.

Frequency Band	Frequency Range (Hz)
LF	0–0.15
HF1	0.15–0.25
HF2	0.25–0.35
HF3	0.35–0.4

**Table 3:** Notations for rule extraction algorithm.

Parameters	Definition
*RRI*	HRV signal sequence
*WAVE*	Wavelet basis functions used
*WL*	Wavelet decomposition level
*WRRI*	HRV signal after frequency division
*RA*	Amplitude value of wave crest and trough
*Amax*	Maximum absolute value of amplitude
*AQ*	Amplitude level quantization sequence
*FI*	Frequent item set
*PR*	Association rule set
*RU*	Standard rule set

**Table 4:** Notations for amplitude level quantitative algorithm.

Parameters	Definition
*AQ_i_*	The *i*-th sequence in the amplitude level quantization sequence
*RU_i_*	The *i*-th rule in the rule set
*MA*	The number of matches
*TC*	Total strength
*FR*	Frequency
*PR*	Polynomial rate
*CO_i_*	Strength of the *i*-th rule in the rule set
*LE*	Total number of grade quantization series
*Lmax*	The maximum length of the matched sequence
*MNl_i_*	The number of sequences of length *i* in the matched quantized sequence
*Acc*	Final recognition accuracy

**Table 5:** Overall performance of the model.

Model	F1-Score	Recall	Specificity	AUC
HER	0.8437	0.8487	0.8602	0.8708
PETSFCNN	0.2962	0.3275	0.4571	0.5735
LSTM-CNN	0.4608	0.5001	0.6688	0.7803
WAVE-SCATTER	0.2953	0.3393	0.5091	0.5946

## Data Availability

A publicly available dataset was analyzed in this study. This data can be found here [https://zenodo.org/record/546113 (accessed on 27 March 2017)].
